# Diagnostic and treatment challenge of unrecognized subacute bacterial endocarditis associated with ANCA-PR3 positive immunocomplex glomerulonephritis: a case report and literature review

**DOI:** 10.1186/s12882-020-1694-2

**Published:** 2020-01-31

**Authors:** D. Bele, N. Kojc, M. Perše, A. Černe Čerček, J. Lindič, A. Aleš Rigler, Ž. Večerić-Haler

**Affiliations:** 1 Department of Cardiology, General Hospital Novo mesto, Novo mesto, Slovenia; 2 Institute of Pathology, Faculty of Medicine, University of Ljubljana, Ljubljana, Slovenia; 3 Medical Experimental Center, Faculty of Medicine, University of Ljubljana, Ljubljana, Slovenia; 4 Department of Cardiology, University Medical Center Ljubljana, Ljubljana, Slovenia; 5 Faculty of Medicine, University of Ljubljana, Ljubljana, Slovenia; 6 Department of Nephrology, University Medical Center Ljubljana, Ljubljana, Slovenia

**Keywords:** Endocarditis, ANCA, ANCA associated glomerulonephritis, ANCA systemic vasculitis

## Abstract

**Background:**

Diagnosis and treatment of either ANCA disease or silent infection-related glomerulonephritis is complicated and is a huge treatment challenge when overlapping clinical manifestations occur. We report a case of ANCA-PR3 glomerulonephritis, nervous system involvement, hepatosplenomegaly and clinically silent subacute infectious endocarditis.

**Case presentation:**

A 57-year-old man with known mitral valve prolaps was admitted for unexplained renal failure with signs of nephritic syndrome, hepatosplenomegaly, sudden unilateral hearing loss, vertigo, malaise, new onset hemolytic anemia and thrombocytopenia. Immunoserology revealed positive c-anti-neutrophil cytoplasm antibody (ANCA)/anti-proteinase 3 (anti-PR3), mixed type crioglobulinemia and lowered complement fraction C3. Head MRI showed many microscopic hemorrhages. Common site of infection, as well as solid malignoma were ruled out. In accordance with clinical and laboratory findings, systemic vasculitis was assumed, although the etiology remained uncertain (ANCA-associated, cryoglobulinemic or related to unrecognized infection). After kidney biopsy, clinical signs of sepsis appeared. Blood cultures revealed *Streptococcus cristatus*. Echocardiography showed mitral valve endocarditis. Kidney biopsy revealed proliferative, necrotizing immunocomplex glomerulonephritis. Half a year later, following intravenous immunoglobulins, glucocorticoids, antibiotic therapy and surgical valve repair, the creatinine level decreased and c-ANCA and cryoglobulins disappeared. A second kidney biopsy revealed no residual kidney disease. Four years after treatment, the patient is stable with no symptoms or signs of vasculitis recurrence.

**Conclusions:**

Here we describe the diagnostic and treatment challenge in a patient with unrecognized subacute bacterial endocarditis associated with ANCA-PR3 immunocomplex proliferative and crescentic glomerulonephritis. In patients with ANCA-PR3 immunocomplex glomerulonephritis and other overlapping manifestations suggesting systemic disease, it is important to recognize and aggressively treat any possible coexisting bacterial endocarditis, This is the most important step for a favorable patient outcome, including complete clinical and pathohistological resolution of the glomerulonephritis.

## Background

Antineutrophil cytoplasmic antibodies (ANCA) against proteinase 3 (PR3) and/or myeloperoxidase (MPO) together with glomerulonephritis have been associated with various disorders. Glomerulonephritis (GN) and vasculitis caused by ANCA is reported to be the most common form of new-onset GN in adults over 50 years. According to recent studies, kidney involvement occurs in 97% of ANCA associated vasculitis [1]. However, ANCA seropositivity can also be found in infection-related GN. Studies report that ANCA seropositivity is diagnosed in 25% of patients with infectious endocarditis-associated GN and in 8% of elderly patients with infection-related GN [2]. Since treatments of ANCA autoimmune disorder and subacute/chronic infection differ drastically, although the clinical manifestation is frequently overlapping, diagnosis and treatment of either ANCA disease or silent infection-related GN is complicated and is a huge treatment challenge.

We report a case of PR 3 ANCA positive immune complex proliferative and crescentic GN in a patient with neurologic involvement and clinically silent subacute/chronic infectious endocarditis and present a unique biopsy-proven report of complete clinical and pathohistological restitution. A brief overview of the literature addressing ANCA positive infectious endocarditis-related GN is also presented.

## Case presentation

**Fig. 1:**
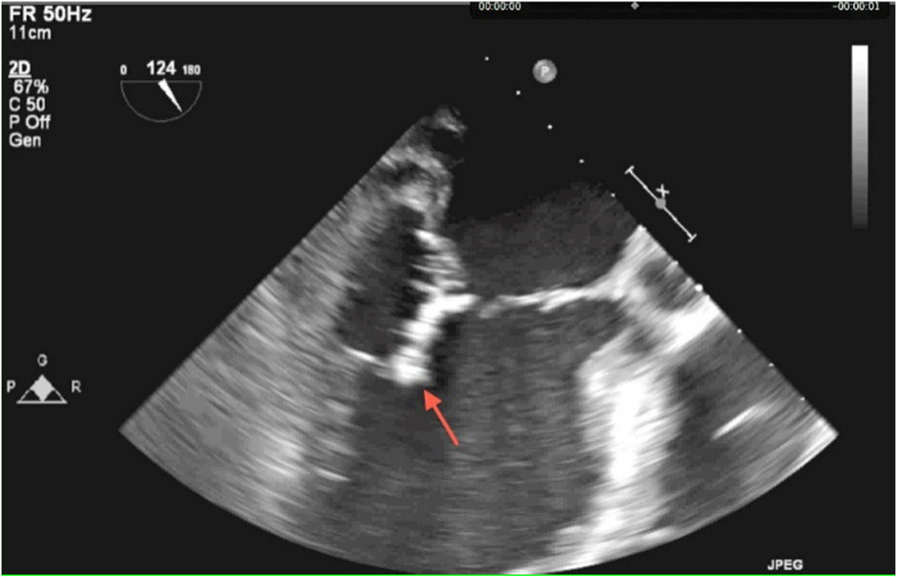
Low esophageal two-chamber view. Shown is large vegetation (arrow) on the posterior leaflet of the mitral valve, which prolapses into the left ventricle during sistole

**Fig. 2:**
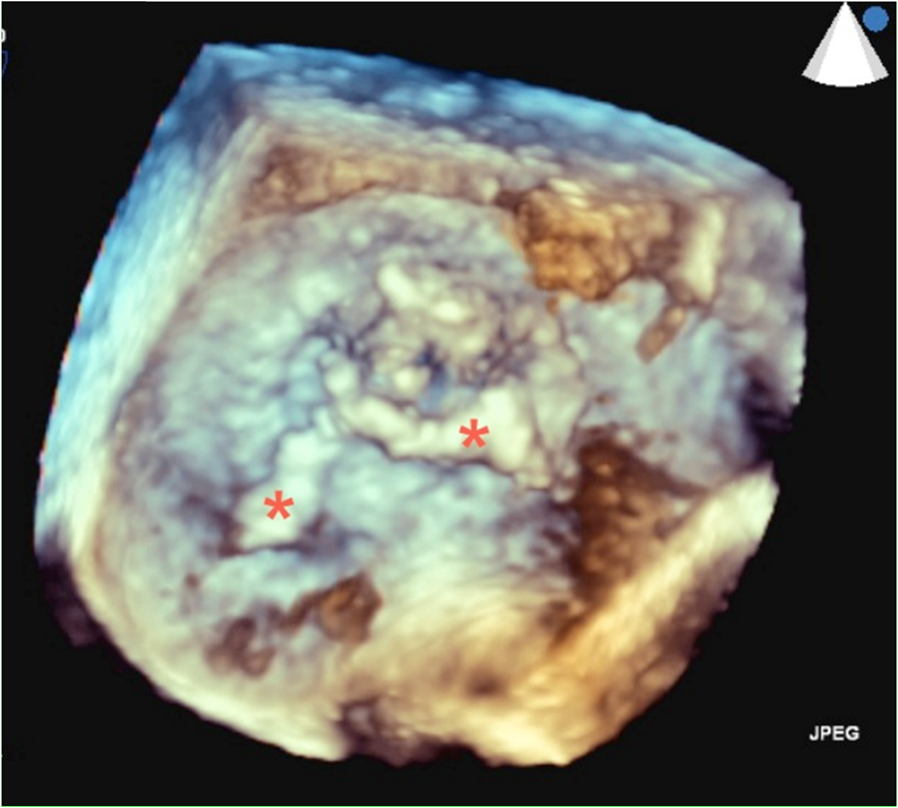
Three-dimensional trans-esophageal view of the mitral valve – viewed from the atrial side. Shown is large branched vegetation (asteriks), which adheres to the P2 scallop of the posterial mitral leaflet

A 57-year old male with a history of neurofibromatosis type 1 and mitral valve prolapse with moderate mitral regurgitation was admitted to a regional hospital due to prolonged dry cough, anemia, hepatosplenomegaly and 6 months non-intentional weight loss. Physical examination revealed many skin neurofibromatomas, a pre-existing holosystolic murmur and enlarged spleen. Laboratory tests showed increased serum creatinine (236 μmol/l), erythrocituria and proteinuria (1.7 g/day), anemia, thrombocytopenia. On admission, immunoserology (HEp-2, ANCA, anti-GBM) was negative. In hospital, he experienced sudden unilateral hearing loss and severe vertigo. Head CT and MRI revealed diffused chronic bilateral microscopic cerebral hemorrhages. Since the clinical course suggested glomerulonephritis (see Table 1) associated with systemic disease, he was referred to the nephrology department. Cystoscopic evaluation performed due to an episode of macrohematuria was unremarkable. Viral hepatitis and HIV serology were negative. Abdominal ultrasound and CT confirmed hepatosplenomegaly with no signs of neoplastic process in the abdomen. Repeated immunoserological tests revealed cANCA/PR3 antibodies (84 IU/mL), low C3 complement fraction and mixed cryoglobulinemia. Electrophoresis of serum and urine was normal. Bone marrow biopsy performed due to haematologic abnormalities and enlarged spleen excluded potential hematologic disorder. Due to neurologic and haematologic disorders, the patient was incapable for a kidney biopsy procedure. Since the diagnosis was uncertain, he was initially treated with intravenous immunoglobulins at a dosage of 2 g/kg body weight. His condition finally improved enough to perform an urgent kidney biopsy. However, immediately after the biopsy, signs of sepsis appeared and a further deterioration of kidney function was observed. *Streptoccus cristatus was isolated from blood cultures*. Transesophageal echocardiography revealed mitral valve endocarditis with very large (3,5 X 0.5 cm), mobile pedunculated vegetation arising from the atrial side of the prolapsing P1-P2 scallops and moderate mitral regurgitation (Figs. 1 and 2). In accordance with these findings, treatment with crystalline penicillin was started.

**Table 1:** Laboratory and serology findings in the patient during a 4-year follow-up

	Reference value	At admission (June 2015)	Prior to cardiac surgery, after introduction of antibiotics, corticosteroid and IVIG (July 2015)	At discharge (September 2015)	At rebiopsy (January 2016)	At reevaluation (June 2016)	At last follow up (June 2019)
Hemoglobin (g/L)	140–180	94	94	112	140	147	149
White cells (10^9/L)	4.0–10.0	3.9	9.7	10.3	10	8.8	7.1
Thrombocytes (10^9/L)	140–340	68	132	200	258	206	180
Urine							
Proteinuria in 24-h urine (g/day)	< 0.15	2.27	nd	0.17	0.74	0.16^a^	0.11^a^
Erythrocituria (number/400x)	< 3	abundant (macrohematuria)	abundant	26	2–4	3	2
sCr (μmol/L)	44–97	341	130	131	117	99	98
CRP (mg/L)	< 5	68	< 5	< 5	< 5	< 5	< 5
C3 (g/L)	0.9–1.8	0.22	0,22		nd	nd	nd
C4 (g/L)	0.1–0.4	0.20	0,20	0.23	nd	nd	nd
Cryoglobulins (mg/L)	< 100	800	688	< 100	nd	< 100	nd
Immunoserology							
cANCA – IF	–	++	No data	+/−	–	–	–
PR3-ANCA-ELISA (IE/mL)	< 10	84	No data	32	4	0	0

^a^estimated daily proteinuria (g/day/1.73m^2^). Legend: *sCr* serum creatinine, *nd* not determined, *IVIG* Intravenous immunoglobulin

**Fig. 3:**
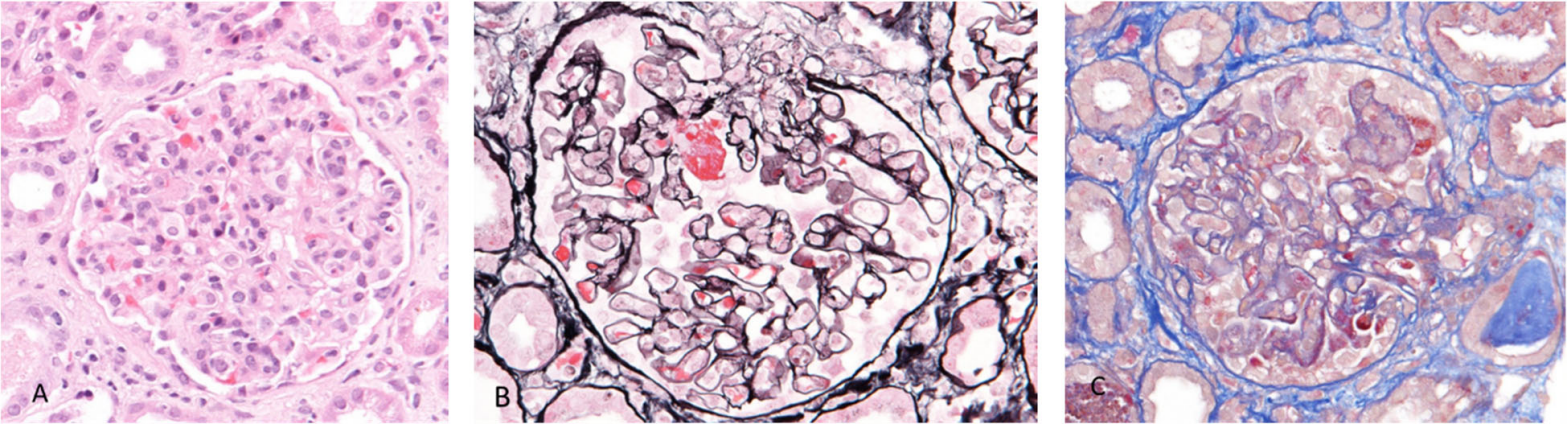
Diffuse proliferative glomerulonephritis (**a**) with focal glomerular necrosis (**b**) and extracapillary crescent formation (**c**) in 13% glomeruli

The pathohistological report of the kidney biopsy revealed uneven proliferative (70%), exudative (32%), necrotizing (10%) and crescentic (13%) glomerulonephritis with mixed inflammatory interstitial infiltration. Immunofluorescence showed glomerular deposits of C3, IgG and IgM, suggesting infection-related immunocomplex GN (Fig. 3). Electron microscopy confirmed electron dense mesangial and segmental subendothelial deposits, without large subepithelial deposits (humps) usually found in infection-related GN.

**Fig. 4:**
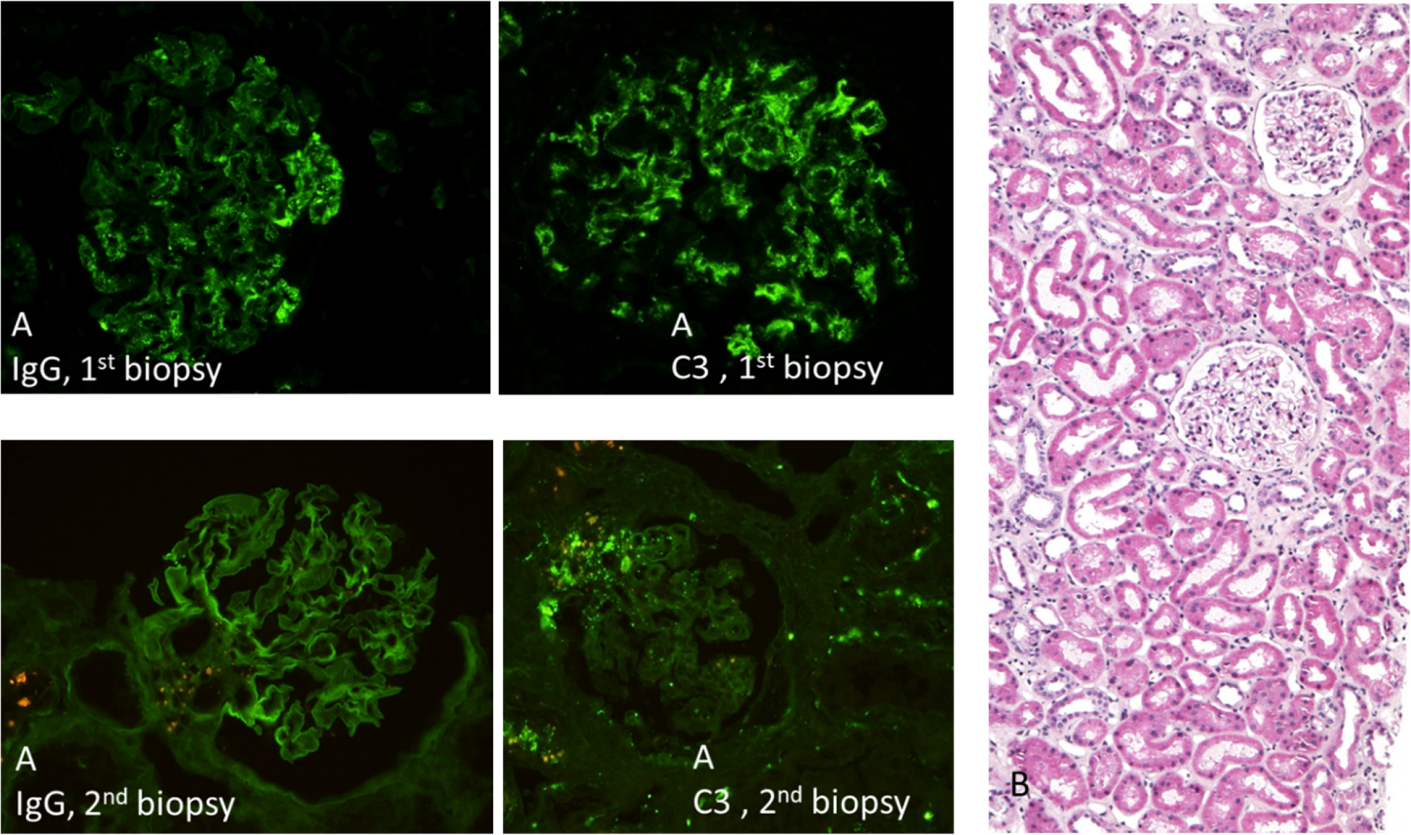
(**a**) Immune complex glomerulonephritis (IgG+, IgM+, C32+) in the 1st biopsy disappeared in the 2nd biopsy (IgG-, IgM + −, C3 + -). **b** Renal parenchyma looked normal

Given our uncertainty of reliably excluding an ANCA driven mechanism of disease, high dose methylprednisolone was introduced (3 pulses 7 mg/kg bw followed by oral methylprednisolone 0.8 mg/kg bw for 1 month with stepwise lowering and exclusion after the second biopsy), which resulted in a gradual improvement of kidney function and general condition. A week later, the patient underwent elective surgical treatment of mitral valve endocarditis. Mitral valve repair with resection of the P1-P2 scallops and mitral valve annuloplasty was performed. After the surgical intervention, his kidney function further improved. At discharge (1 month after the mitral valve operation) his serum creatinine (131 umol/l) and PR3-ANCA titer (32 IU/mL) were still increased, while blood cryoglobulin level had normalized (< 100 mg/l) (Table 1). In addition, abdominal ultrasound showed a reduction of spleen size, and the vertigo had disappeared. However, unilateral hearing loss remained. Six months after the first biopsy, laboratory tests and a second biopsy were performed. Improvement of kidney function (serum creatinine 100 μmol/l), negative PR3 ANCA levels, restituted serum complement levels, and persistent minimal glomerular erythrocituria were observed. The second kidney biopsy revealed complete kidney resolution, including an absence of immune deposits (Fig. 4). Today, 4 years after the 1st biopsy the patient has persistent unilateral hearing loss but stable renal function (serum creatinine 98 umol/l), negative PR3 ANCA and cryoglobulins levels, and unremarkable urine sediment (Table 1).

## Discussion and conclusions

The principal aim of this case report is to highlight the diagnostic and treatment challenge in patients with ANCA-PR3 GN, nervous system involvement, hepatosplenomegaly and clinically silent subacute/chronic infectious endocarditis.

It is nowadays well established that ANCA is a clinically relevant diagnostic marker for systemic ANCA vasculitis, also predictive of renal disease activity [1, 3]. However, recent publications report that ANCA seropositivity (MPO or PR3) also develops in adults with infection-related GN [2]. One of the largest studies addressing the association of ANCA vasculitis and chronic infection discovered that the majority of patients with previously confirmed ANCA vasculitis accompanied with chronic infections were finally re-diagnosed as subacute bacterial endocarditis [4]. In addition, recent cohort studies discovered that 24–33% of patients with documented subacute bacterial endocarditis were found to have ANCA positivity (predominantly PR3) and immune complex GN [5–8]. Since ANCA positive subacute/chronic infections can mimic the clinical presentation of idiopathic ANCA associated vasculitis (AAV), the differential diagnosis and treatment of these two diseases is very challenging. In order to diagnose and treat patients with ANCA positive GN optimally, two main questions need to be answered. First, how to differentiate patients with unrecognized subacute or chronic infection from idiopathic AAV and second, what is the optimal treatment option in a case of uncertainty. There are currently no guidelines on the treatment of subacute bacterial endocarditis associated with ANCA positive GN. In order to answer these two questions, we searched and reviewed the literature describing patients with ANCA-positive GN accompanied by infectious endocarditis.

### How can we differentiate ANCA-positive patients with silent subacute/chronic infection from idiopathic AAV?

In 2010, Bonaci-Nikolic et al. [4] investigated which markers might be useful in the diagnosis and prognosis of ANCA associated infections. They discovered that AAV patients and ANCA-positive patients with infection displayed a high frequency of arthralgia/myalgia, fever and weight loss. No distinctions in various skin manifestations and kidney involvement were observed.

However, patients with AAV had significantly more ear/nose/throat involvement, pulmonary manifestations, pulmonary-renal syndrome and nervous system manifestations, while ANCA-positive patients with infections showed a higher frequency of spleen and/or liver enlargement and new onset heart murmurs [4]. Importantly, patients with infections more frequently expressed dual ANCA (high PR3/low MPO) positivity, accompanied by the presence of mixed cryoglobulins, ANA, hypocomplementemia, anti-cardiolipin and anti-beta2 GPI antibodies [4]. Interestingly, in 2012, Forbes et al. suggested diagnostic aids in order to differentiate between ANCA positive infectious endocarditis and AAV. Constitutional symptoms such as active urinary sediment, decreased GFR, fever, skin involvement and increased levels of inflammatory markers might be observed in both conditions, while clinical manifestations, including splenomegaly, thrombocytopenia, hypocomplementemia, immune complexes, other positive autoantibodies, low titer ANCA/ELISA negative and other organ involvement might be associated predominantly with infectious endocarditis [9]. Nevertheless, recent cohort studies have shown that neurologic involvement [8] or micro bleedings diagnosed by cerebral MRI can be seen not only in AAV patients but also in patients with infectious endocarditis-associated GN (emboli) [7]. In addition, pulmonary inflammatory granulomas, which are more frequently seen in idiopathic AAV patients, have also been reported in ANCA-positive infectious endocarditis-associated GN [10]. On the other hand, fever (a typical sign of infection) can be absent in some patients with ANCA positive infectious endocarditis-associated GN [11, 12]. In particular, the elderly population may lack specific signs of infection and fever might occur in only 70–80% of patients [2]. Moreover, a negative blood culture seen in patients with infectious endocarditis-associated GN represents an additional diagnostic and therapeutic challenge [13]. Thus, when signs of infection are nonspecific, especially in the absence of pyrexia, infectious endocarditis accompanying ANCA positive GN may be misdiagnosed, resulting in inappropriate or even fatal treatment.

Diagnosis of ANCA-associated vasculitis is based not only on the presence of clinical manifestations and PR3-ANCA or MPO-ANCA but also on histopathological and immunopathological findings. It is widely accepted that pauci-immune necrotizing and crescentic GN is a typical pattern of AAV [1], while infection-related GN is typically immune complex with various immune deposits (C3, IgG, IgM, IgA, C1q). The most frequently detected pattern of glomerular injury in infection-related GN includes endocapillary hypercellularity and exudation of neutrophils, whereas crescentic and necrotizing GN might be rarely observed [2, 14]. Interestingly, although the widely accepted histological pattern of infectious endocarditis-related GN reported in renal medicine and pathological textbooks is diffuse or focal proliferative and exudative GN with glomerular exudation of neutrophils [2], recent studies have indicated that the histological as well as immunologic pattern in the case of infectious endocarditis-associated GN might have changed over the last decades. The largest cohort of 49 infectious endocarditis-associated GN diagnosed by kidney biopsy revealed that nowadays (in the era of antibiotics), patients with infectious endocarditis-associated GN in most cases develop crescentic and necrotizing GN (53%) followed by proliferative GN (37%) and mild mesangial hypercellularity GN (10%). Interestingly, classic infection-related subepithelial hump-like deposits were rarely observed (14%) in infectious endocarditis-associated GN [5]. Similarly, a review of case reports on ANCA-positive patients with infectious endocarditis-associated GN reported various histological patterns of GN in renal biopsy, such as extracapillary GN with immune deposits, segmental and focal necrotizing GN, endocapillary GN with immune deposits, interstitial nephritis, chronic sclerotic GN, and both endocapillary GN and interstitial nephritis [8]. Although most patients had detectable immune deposits, C3 being most prominent, pauci-immune GN in patients with PR3-ANCA bacterial endocarditis has also been observed [15, 16]. Taken together, although the histological appearance of infectious endocarditis-associated GN shows great variability, it is important to take into consideration that immune deposits usually indicate infection-related GN, regardless of ANCA positivity. A renal biopsy may therefore enable additional help in differentiating between infection-related, including infectious endocarditis- associated GN, and idiopathic AAV.

### What is the optimal treatment option in doubtful cases?

Our patient had a heterogenous clinical presentation (renal impairment, ANCA-PR3 seropositivity, hypocomplementemia, absence of clear signs of infection) and was treated at first with intravenous immunoglobulins, followed by steroids and antibiotics. Such an approach has also been used by others in similar cases [11, 12]. Although infective endocarditis (even when associated with kidney injury) can be optimally treated with appropriate antibiotics without surgical repair [17], in our patient, reconstruction of the mitral valve was necessary due to large, floating vegetation and was performed immediately after the patient’s health stabilized. Nevertheless, complete recovery of the patient with clinical (including biochemistry and serology findings) and histologic remission of renal injury and normalization of heart function was achieved without any side effects of immunosuppressive therapy. After complete remission, corticosteroid therapy was withdrawn (after the second biopsy). Four years after treatment, the patient is stable with no symptoms or signs of disease recurrence.

Literature regarding the appropriate treatment of patients with ANCA positive infectious endocarditis-associated GN is currently not consistent. Some propose treatment with antibiotics alone [10], others propose a combination of antibiotics and steroids [11, 18–20] and a third group suggest surgery followed by antibiotics without steroids [21]. Interestingly, in 1998 Haseyama et al. [19] proposed that patients with ANCA positive infectious endocarditis-associated GN and low titers of PR3 ANCA (e.g., below 25 IU/ml) can be treated with antibiotics alone, while patients with higher titers of PR3 ANCA antibodies (e.g. above 50 IU/ml) or when they do not respond to antibiotic treatment within a proper period of time, are advised to use either antibiotics and corticosteroids or antibiotics and immunosuppressants [19]. Although there are also reports that steroids do not have any role in the treatment of infectious endocarditis-associated GN, a recent case report clearly showed that in a patient with ANCA positive infection-related GN, antibiotic treatment alone was not effective. Despite the risk of aggravating the infection, only the combination therapy of corticosteroid and antibiotic improved the disease [22]. Nevertheless, due to the paucity of reports, the addition of corticosteroids to antibiotics and operative treatment should probably be individualized in the presence of bacterial endocarditis.

Taken together, the literature shows that the clinical and histological manifestation of ANCA-positive infectious endocarditis-associated GN can be variable, making differential diagnosis between infectious endocarditis-associated GN and idiopathic AAV very difficult, particularly in cases of infection with a clinically silent course. The presented case of subacute infectious endocarditis associated with c-ANCA/anti-PR3 and immune complex GN, and many overlapping clinical manifestations suggesting systemic disease, underlines the importance of recognizing and aggressively treating any possible coexisting bacterial endocarditis or other chronic infection in ANCA positivity. It is the most important step for complete and stable remission.

## Data Availability

Data sharing is not applicable to this article as no datasets were generated or analysed during the current work (case report). Additional (detailed) information on patients’ evaluation and treatment are available from the corresponding author on reasonable request.

## Abbreviations

AAV: ANCA associated vasculitis
ANCA: Antineutrophil cytoplasmic antibodies
c ANCA: cytoplasmic ANCA (refers to immunofluorescent pattern)
GN: Glomerulonephritis
MPO: Myeloperoxidase
PR3: Proteinase 3
